# G Protein-Coupled Receptors: What a Difference a ‘Partner’ Makes

**DOI:** 10.3390/ijms15011112

**Published:** 2014-01-16

**Authors:** Benoît T. Roux, Graeme S. Cottrell

**Affiliations:** 1 Department of Pharmacy and Pharmacology, University of Bath, Bath BA2 7AY, UK; E-Mail: b.roux@bath.ac.uk; 2 Reading School of Pharmacy, University of Reading, Reading RG6 6UB, UK

**Keywords:** GPCR, interacting protein, chaperone, escort protein, accessory protein, signaling modulation, trafficking

## Abstract

G protein-coupled receptors (GPCRs) are important cell signaling mediators, involved in essential physiological processes. GPCRs respond to a wide variety of ligands from light to large macromolecules, including hormones and small peptides. Unfortunately, mutations and dysregulation of GPCRs that induce a loss of function or alter expression can lead to disorders that are sometimes lethal. Therefore, the expression, trafficking, signaling and desensitization of GPCRs must be tightly regulated by different cellular systems to prevent disease. Although there is substantial knowledge regarding the mechanisms that regulate the desensitization and down-regulation of GPCRs, less is known about the mechanisms that regulate the trafficking and cell-surface expression of newly synthesized GPCRs. More recently, there is accumulating evidence that suggests certain GPCRs are able to interact with specific proteins that can completely change their fate and function. These interactions add on another level of regulation and flexibility between different tissue/cell-types. Here, we review some of the main interacting proteins of GPCRs. A greater understanding of the mechanisms regulating their interactions may lead to the discovery of new drug targets for therapy.

## 1. Introduction

The largest family of cell-surface receptors is the superfamily of the G protein-coupled receptors (GPCRs). The GPCR family is characterized by its seven transmembrane spanning domains and represents the largest class of drug targets. There are more than 800 known GPCRs in the human genome, which are involved in virtually every physiological process [1]. GPCRs can be divided into five subfamilies: the rhodopsin (originally called A or I), secretin (B or II), glutamate (C or III), adhesion and frizzled/taste2 [1]. GPCRs can be activated by a wide range of stimuli, such as photons of light, odorant molecules, peptides, hormones and lipid molecules [2].

GPCRs play a central role in cell signaling and it is vital for organisms to maintain the right balance between activation and desensitization. Therefore, GPCRs must be carefully regulated by the many different cell systems in which they are present. In order for signal transduction to be initiated by a GPCR, it must be present at the cell-surface. This prerequisite provides a mechanism for regulation. Only a few GPCRs are known to possess an identified *N*-terminal endoplasmic reticulum (ER)-export signal, which promotes trafficking to the cell-surface. Furthermore, exit from the ER also requires correct folding of GPCRs, which is promoted by chaperone proteins. Improper folding leads to retention of GPCRs in ER and/or targeting to the proteasome for degradation [3–5]. The concept of chaperones was first proposed in 1987, to define a class of proteins involved in the correct folding of newly synthesized proteins [6]. Heat shock proteins (HSPs) are commonly considered as the central components of this system. In general, HSPs recognize hydrophobic domains exposed by unfolded proteins and prevent inappropriate association or aggregation of these domains, subsequently promoting the correct folding of these proteins [7]. HSPs were originally identified as being upregulated after heat shock or other stresses promoting survival of the organism [8]. Such stress events can potentially modify a protein’s properties, promoting an “unfolded” protein state that has a higher probability of reaching an irreversible unfolded state or of forming aggregates that can eventually lead to cell death. The HSPs consist of many structurally unrelated protein families [9], where the HSP70 and HSP40 family are the most characterized. HSP40 are co-factors of the HSP70 that comprise a J-domain allowing the recruitment and the ATPase activity of the HSP70 [10].

Small cell-permeable compounds known as pharmacological chaperones are able to rescue mutation-mediated misfolding of vasopressin 2 (V_2_) receptor, involved in diabetes [11] and rhodopsin 1 (Rh1) receptor, involved in retinitis pigmentosa [12]. These pharmacological chaperones are believed to bind to newly synthesized receptors, stabilizing their structure to enable normal trafficking to the cell-surface. As well as quality control for exit from the ER, interactions with specific molecular chaperones and GPCRS is known to promote trafficking to the cell-surface, maturation and even modulate the signaling of these receptors.

GPCRs transfer extracellular signals across the plasma membrane to intracellular effectors via G proteins. G proteins belong to the GTPase family and consist of three protein subunits, an α-subunit, a β-subunit and a γ-subunit. The β- and γ-subunits form a stable dimeric complex referred as the βγ-subunit. The binding of the agonist to the GPCR induces a conformational change of the receptor promoting activation of heterotrimeric G proteins by interaction with the GPCR. Upon activation, the Gα-subunit dissociates from the βγ-subunit complex. Both Gα-subunit and the βγ-subunit complex are then free to activate downstream effectors [13,14]. Gα-subunits are divided into four families based on similarity of the α-subunits: Gα_s_, Gα_i/o_, Gα_q/11_ and Gα_12/13_. The Gα_s_ and Gα_i/o_ families activate or inhibit the activity of adenylate cyclase resulting in an increase or decrease of cyclic AMP (cAMP) production, respectively. The Gα_q/11_ family stimulates phospholipase C (PLC) that hydrolyzes phosphatidylinositol phosphates leading to intracellular Ca_2+_ mobilization (from the ER) and activation of protein kinase C (PKC). Finally, the Gα_12/13_ family activates small GTPases including the Rho family (e.g., RhoA). These second messengers then activate several intracellular pathways to modulate cell function [13,15].

Activation of GPCRs triggers not only activation of G proteins, but also other cellular events that lead to a rapid attenuation of receptor responsiveness, a process termed desensitization. Signaling of activated GPCRs at the cell-surface must be rapidly terminated in order to prevent uncontrolled signaling. The first step in desensitization is phosphorylation of the receptor [16,17], mainly by a kinase family named GPCR kinases (GRKs) [18,19]. Since their discovery, GRKs have been shown to play a central role in the desensitization of many GPCRs [20]. Further studies identified another protein family that was only able to bind phosphorylated GPCRs, called arrestins. The arrestin family of proteins comprises four members, arrestin1-4, also known as visual arrestin, β-arrestin1, β-arrestin2 and cone arrestin, respectively [21,22]. Unlike arrestin 1 and 4, the β-arrestins are ubiquitously expressed and play a conical role in the desensitization of many GPCRs. Interaction of β-arrestins with activated GPCRs promotes the uncoupling of the GPCR from G proteins, terminating the G protein-dependent signaling initiated at the cell-surface. In addition to termination of signaling, β-arrestins also facilitate internalization of GPCRs and act as a molecular scaffold recruiting signaling proteins to internalized GPCRs in endosomes to activate G protein-independent signaling cascades. The roles of GRKs and β-arrestins in the trafficking and signaling of GPCRs has been extensively studied and are reviewed elsewhere [23–27].

After internalization, the fate of GPCRs depends on both the cell type and on the type of receptor. Typically, following agonist-induced internalization, GPCRs are efficiently recycled back to the cell-surface. However, many GPCRs are trafficked to a degradative pathway and proteolytically destroyed in the lysosome, a process called down-regulation. Again, the mechanisms involved in this process have been the subject of many studies and they are reviewed elsewhere [25,27,28].

Recent studies have challenged the traditional concepts of GPCR activation, where one ligand interacts with one receptor to couple to one G protein and thereby initiating signaling. In fact, we now understand that different ligands can interact with same GPCR to stabilize different receptor conformations. This ligand-specific conformation of the GPCR then promotes unique signaling properties. During their “life”, GPCRs have been shown to interact with many proteins. These interactions are important to regulate GPCRs and maintain the correct balance between signaling and desensitization. There is now increasing evidence that many GPCRs interact with specific interacting proteins that alter their expression, function and fate. These proteins are often referred as “accessory” or “escort” proteins and how the specific interactions with these accessory proteins lead to critical changes in GPCR expression and signaling constitutes the topic of this review. It must be noted that many GPCRs have been shown exist as homo- or hetero-dimers (or even higher order oligomers) and that these interactions are important for modulating the functional properties of GPCRs. Therefore, GPCRs themselves can be considered as accessory proteins. However, only non-GPCR accessory proteins will be discussed in this review, but for more details see [44,45]. Here, we review a non-exhaustive list of the major GPCR-interacting proteins, and merge some old and new concepts in order to understand these mechanisms.

## 2. Interacting Proteins that Modulate Cell-Surface Localization of GPCRs

**Figure 1.**
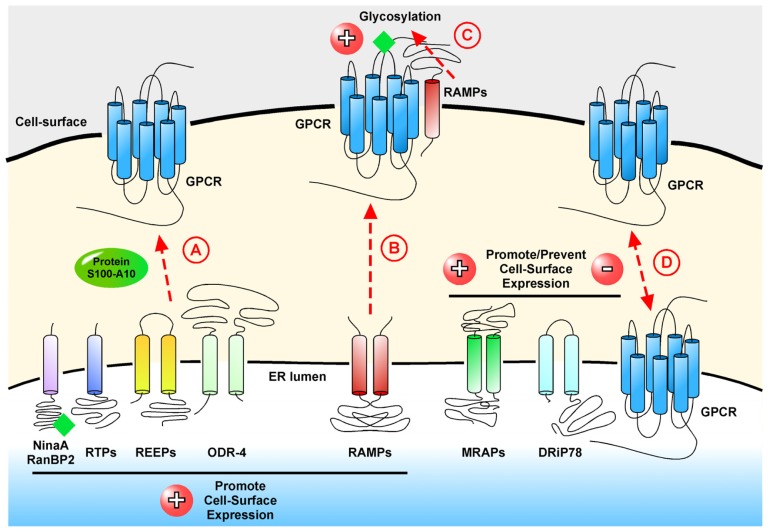
Schematic representation of the roles of GPCR interacting proteins in the localization of GPCRs at the cell-surface. (**A**) NinaA, RanBP2, RTPs, REEPs, ODR-4 and protein S100-A10 facilitate cell-surface localization by promoting correct folding and protein trafficking; (**B**) Receptor activity-modifying proteins (RAMPs) act as chaperones and traffic with GPCRs to the cell-surface and (**C**) promote glycosylation of the GPCR; (**D**) Melanocortin 2 receptor accessory proteins (MRAPs) and dopamine-receptor-interacting protein of 78 kDa (DRiP78) either promote or prevent cell-surface localization depending on the particular GPCR. 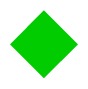 represents glycosylation.

The appropriate expression and localization of GPCRs is essential so that cells can maintain proper communication pathways within an organism. Indeed, dysregulation of process that control if a GPCR reaches the cell-surface and if it internalizes or is down-regulated properly can lead to aberrant cell signaling and potentially lead to disorders and disease. Studies have revealed that certain GPCRs require specific factors to properly regulate their trafficking to or from the cell-surface (Table 1, Figure 1).

### 2.1. ninaA and RanBP2: Modulators of Opsin Receptors

The *Drosophila melanogaster* gene *ninaA* (neither inactivated nor after potential A), was initially found to encode a protein required for the biogenesis Rh1 receptor [29,30]. ninaA was shown to share sequence homology and function with cyclophilin A, a protein that exhibits a peptidyl-prolyl cis-trans isomerase activity and is involved in the folding of proteins [30,46]. Knockout studies of *ninaA* showed that the Rh1 receptor is retained in ER and unable to traffic to the cell-surface in absence of ninaA [31]. The Rh1 receptor and ninaA form a stable complex *in vivo* and mutations in ninaA are sufficient to promote a significant reduction in the presence of Rh1 receptors at the cell-surface [31,47]. The observation that the Rh1 receptor and ninaA form a stable complex and co-localize in different vesicles within the cytoplasm, suggests that ninaA is an escort protein that is required for the proper folding and trafficking of Rh1 receptors [31,47]. In fact, ninaA was the first “private” chaperone described for a GPCR.

RanBP2 (Ran binding protein 2) is the mammalian homologue of ninaA, which is also specifically expressed in photoreceptor cells. RanBP2 interacts with red/green opsins and is believed to have similar function that its homologue. However, unlike ninaA, the cyclophilin domain of RanBP2 does not directly bind to the opsin receptor, but augments binding to its Ran binding domain 4 (RBD4) [32].

### 2.2. RTPs and REEPs: Traffic of the Odorant Receptors

Even though the existence of mammalian odorant receptors (ORs) has been known for over 20 years [48], studies have been hampered, simply because of their lack of expression at the cell-surface of heterologous cells. The reason behind this is that when expressed in model cell systems they are retained in the ER and then degraded by the proteasome [49,50]. These observations raised the question that perhaps ORs require specific accessory proteins to regulate their expression at the cell-surface. A study using *Caenorhabditis elegans* showed that the transport of ORs to the cilia of olfactory neurons required the expression and association of ORs with a transmembrane protein called odorant protein 4 (ODR-4) [51]. It has been subsequently found that co-transfection of rat ORs with ODR-4 in immortalized olfactory sensory neurons from rat, called odora cells [52] and CHO cells enhances the transport and expression of ORs at the cell-surface [53]. These studies suggested that olfactory neurons have a selective molecular machinery that promotes expression of ORs at the cells surface [53,54]. In an attempt to identify new accessory proteins involved in the trafficking of ORs to the plasma membrane, single olfactory neurons and neurons from the vomeronasal organ of mouse were screened for genes that encode membrane-associated proteins. Potential candidates were then cloned and co-transfected in HEK293T cells with a mouse OR, MOR203-1 [33]. This study identified two protein families able to facilitate expression of MOR203-1 at the cell surface. Receptor transporting protein 1 and 2 (RTP1, RTP2) both strongly induced expression of several ORs at the cell-surface. The receptor expression enhancing protein 1 (REEP1) appeared to also promote cell-surface expression, but to a much smaller extent compared to RTP1 and 2 [33].

Interestingly, these accessory proteins are specifically expressed in olfactory neurons, not even in testis, where a subsets of ORs are expressed [55,56], suggesting the presence of a cell-specific mechanism that regulates transport of ORs to the cell-surface in testis. Indeed, other members of the RTP and REEP families show a more widespread distribution than the olfactory sensory neurons. For instance, RTP3 is expressed in liver, lung, and testis, whereas RTP4 and several REEP gene are expressed in amygdala, bladder, bone marrow, colon, kidney, liver, lung, lymph node, macrophages, mammary gland, melanocytes, nasopharynx, pituitary, prostate, retinal pigment epithelium, spinal cord, spleen, testis, thymus and uterus [34]. However, RTP3 and RTP4 have not been shown to promote cell-surface expression of ORs in testis, but to another class of GPCRs, the bitter taste receptors, TAS2Rs [34].

Although, δ and μ opioid receptors (δ and μ receptor) are expressed at the cell-surface when transfected separately in HEK293 cells (e.g., [57]), it has been reported that when they are co-transfected they form a heterodimer that is, retained in the trans-Golgi apparatus, ubiquitinated and degraded by the proteasome [35]. However, expression of RTP4 enhances cell-surface localization of δ-μ receptor heterodimer and subsequently prevents its ubiquitination and degradation [35].

Recently, sequence comparisons showed that REEP shares structural homology with the plant protein HVA22s and the yeast protein YOP1P both of which belong to the Ypt-interacting protein (Yip) family. Therefore, REEP was reclassified as part of this family [58]. Yips are transmembrane proteins that bind to Rab proteins. Their exact functions remain unknown; however Yip3 members have been showed to be involved in the vesicular transport [59]. Studies have shown that REEP1 and REEP5 (also known as DP1) are involved in shaping the ER by linking microtubule fibers to the ER [58,60]. A recent study looking at the role of REEP in the trafficking of α_2A_- and α_2C_-adrenoceptors showed that REEP1-2 and 6 enhance the cell-surface expression of α_2C_-adrenoceptors, but not α_2A_-adrenoceptors, by increasing the capacity of ER cargo, thereby allowing more receptors to reach the cell-surface [61]. Unlike RTP1, REEP1-2 and 6 are only present in the ER, do not traffic to the plasma membrane and specifically interact with the minimal/non-glycosylated forms of α_2C_-adrenoceptors through an interaction with its *C*-terminus [33,61]. Thus, REEPs seem to function as important modulators of the ER, rather than specific GPCRs-interacting proteins.

Even though much has been learned about the role of RTP family proteins in the trafficking of GPCRs, the exact mechanisms by which RTPs promote trafficking remain poorly understood. For example, it is not known if RTPs facilitate the transport of GPCRs through specific vesicles/cargo, as has been described for REEPs. Moreover, given that RTP1 and ORs form a complex at the cell-surface [33], it is possible to imagine that RTPs are able to modulate the ligand binding of GPCRs. However, RTPs do not seem to affect ligand specificity of two mouse ORs [33]. Nonetheless, this possibility cannot be excluded for other GPCRs. The wide distribution of RTPs implies that they are likely to be involved in the regulation of yet unidentified GPCRs.

### 2.3. Protein S100-A10 and Its Role in Depression

Protein S100-A10 (also known as p11) was first identified as part of a heterotetrameric complex with annexin A2, a protein involved in the linkage of the actin cytoskeleton to membrane-associated proteins [62]. Protein S100-A10 was subsequently found to interact with many other proteins, including ion channels (e.g., Na_V_1.8 sodium channel and TASK1 potassium channel [63,64]), proteolytic enzymes (e.g., cathepsin B and the plasminogen activator [65,66]) and GPCRs (e.g., 5-Hydroxytryptamine (HT) _1B_ and 5-HT_4_ receptors [36,37]). Protein S100-A10 belongs to the S100 EF-Hand protein family, the largest group of the superfamily of proteins containing a Ca^2+^-binding domain called EF-Hand motif [67]. S100 are small acidic proteins (10–20 kDa) that exist as homo- and heterodimers, where each monomer contains two EF-Hand motifs. However, protein S100-A10 is unique in the fact that it has two mutations in its two EF-Hand motifs, which makes it unable to bind Ca^2+^ [67]. Protein S100-A10 is mainly expressed in regions of the brain that are involved in the pathophysiology of depression, such as the nucleus accumbens, cerebral cortex and hippocampus [68–70]. Therefore, it was hypothesized that protein S100-A10 may play a role in depression (reviewed in [71]). The pathophysiology of depression remains unclear, however, dysregulation of 5-HT receptors (5-HTRs) have shown to be important in the development of the disease [72].

Studies looking for a link between protein S100-A10 and 5-HTRs observed that protein S100-A10 binds to the third intracellular loops of 5-HT_1B_R and 5-HT_4_R [36,37]. They also reported that when 5-HT_1B_R and 5-HT_4_R were transfected in COS-7 cells, their cell-surface expression was increased when co-transfected with protein S100-A10. Likewise, protein S100-A10 has been shown to potentiate the effects of 5-HT on 5-HT_1B_R- and 5-HT_4_R-mediated cAMP production, whereas protein S100-A10-knockdown reduced the binding of 5-HT to the same receptors. However, the exact mechanisms by which protein S100-A10 regulates transport of 5-HT_1B_R and 5-HT_4_R to the cell-surface remain to be determined.

Interestingly, patients who had suffered from unipolar major depression disorder were found to have lower levels of both protein S100-A10 mRNA and protein, similar observations made in a mouse model of depression (H/Rouen mice). Conversely, treatment with two antidepressants, imipramine (inhibits 5-HT reuptake) and tranylcypromine (a monoamine oxidase inhibitor that inhibits 5-HT degradation), significantly increased levels of protein S100-A10 mRNA [36]. In behavioral studies wild-type mice showed increased mobility in the forced swim and tail suspension tests following treatment with anpirtoline (5-HT_1B_R agonist) and RS67333 (5-HT_4_R agonist) reflecting a decreased in the depression state [73,74]. However, protein S100-A10-knockout mice exhibited a reduced mobility in similar tests, indicating that these mice present a depression-like phenotype [36,37]. Therefore, protein S100-A10 may play an important role in depression, through its ability to modulate 5-HTR expression at the cell-surface. Protein S100-A10 has been shown to bind to a wide variety of proteins (e.g., annexin A2, peptidases and ion channels), therefore it is likely that protein S100-A10 and other members of the S100 protein family also regulate the expression and/or signaling of other GPCRs.

### 2.4. GASPs and SNXs: Interacting Proteins in GPCR Down-Regulation

**Figure 2.**
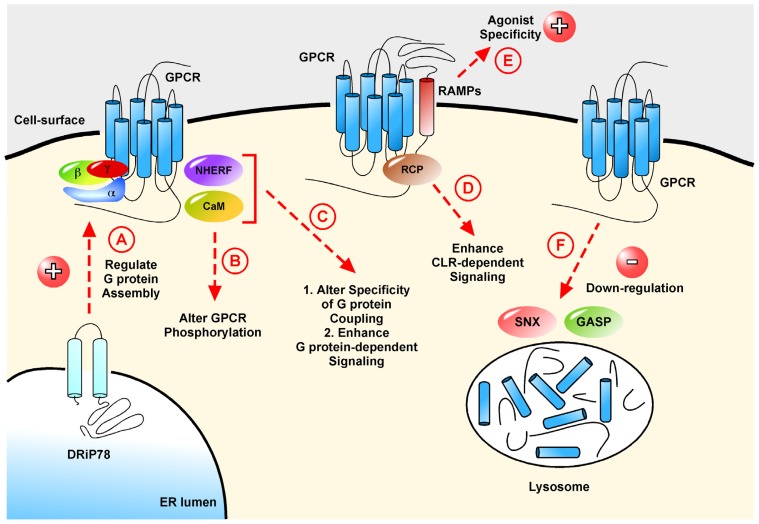
Schematic representation of the roles of GPCR interacting proteins in the signaling and trafficking of GPCRs from the cell-surface. (**A**) DRiP78 coordinates the assembly of the G protein•GPCR complex to ensure proper GPCR signaling at the cell-surface; (**B**) Following activation of certain GPCRs, CaM regulates GPCR phosphorylation; (**C**) Calmodulin (CaM) and Na^+^/H^+^ exchanger regulatory factor (NHERF) both act to specify G protein coupling and enhance G protein-dependent signaling; (**D**) Receptor component protein (RCP) specifically interacts with CLR•RAMP complexes to enhance signaling; (**E**) At the cell-surface, RAMPs interact with GPCRs to confer agonist specificity; (**F**) GASPs and SNXs interact with activated GPCRs to promote efficient down-regulation and thereby permanently terminate signaling.

The removal of GPCRs from the cell-surface is another mechanism to ensure termination of signaling. This process is termed endocytosis and controls and regulates the entry and exit of small and large molecules. Endocytosis is influenced by GRKs, β-arrestins and ubiquitin (a small regulatory protein involved in the degradation of proteins) (reviewed in [23,28,75]). However, the down-regulation of GPCRs has also been shown to depend on other accessory proteins (Table 1, Figure 2).

The GPCR-associated sorting protein (GASP) family comprises a family of 10 proteins that interact with a broad spectrum of GPCRs [76]. The first family member GASP1 was identified as a binding partner of the δ receptor [38]. The interaction between δ receptor and GASP1 promotes traffic of the receptor to lysosomes, inducing its degradation. Overexpression of cGASP1 (a dominant negative of GASP1 consisting of the final 497 residues of the protein) or mutation of the interaction domain of GASP1 allows the δ receptor to escape from lysosomal degradation and promotes recycling of the receptor. GASP1 also interacts with the *C*-terminal tails of dopamine 2 (D_2_) receptors [39], μ and κ opioid receptors, β_1_-adrenoceptors and calcitonin receptors (CTR) [40]. GASP1 is widely distributed, but is predominantly expressed in the central nervous system [40]. Interestingly, GASP1 has also been found to bind β_2_-adrenoceptor, a receptor that usually undergoes efficient recycling, a fact that suggests that GASP1 may be involved in the sorting of β_2_-adrenoceptor for down-regulation which occurs following chronic stimulation [38]. More recently, co-immunoprecipitation together with mutagenesis studies reveal a highly conserved and repeated GASP motif (SWFW) that determines the interaction between GASPs and GPCRs [77]. Indeed, the discovery of this GASP motif lead to the identification of two different classes of GASPs: GASP1-5 contain this motif and generally bind strongly to GPCRs (GASP1-5); whereas GASP6-10 does not possess this motif and only weakly bind to GPCRs [77].

Sorting nexins (SNX) are another protein family involved in the endosomal sorting of GPCRs to lysosomes. These membrane-associated proteins are characterized by the presence of a PX domain (named after NADPH oxidase domains of p40phox and p47phox), consist of approximately 100–130 amino acids and have the ability to bind various phosphatidylinositol phosphates [78]. SNXs were first implicated in the sorting of the epidermal growth factor receptor (ErbB1), a receptor tyrosine kinase. Stimulation of ErbB1 with epidermal growth factor promotes binding of SNX-1 to the receptor and induces degradation of ErbB1. Degradation of ErbB1 was enhanced by overexpression of SNX-1 [79]. Proteinase-activated receptor 1 (PAR_1_) was the first GPCR identified to be regulated by interaction with SNX-1. The *C*-terminal tail of SNX-1 acts as a dominant negative and blocks agonist-induced trafficking of PAR_1_ to endosomes [41], suggesting that SNX-1 was required for the trafficking of PAR_1_ to lysosomes. This requirement was later confirmed using siRNA to deplete cells of endogenous SNX-1 [42]. SNX-1 has been shown to bind other GPCRs, including the oxytocin receptor and δ receptor, both of which are known to undergo lysosomal sorting [43]. Similar to GASP1, SNX-1 has been found to bind to members of the tachykinin receptor family (e.g., neuorkinin 1 receptor) and acetylcholine receptor (muscarinic) family, which are known to recycle back to the cell-surface [43], suggesting a role for SNX-1 in down-regulation of GPCRs following chronic exposure to agonist.

## 3. Interacting Proteins that Modulate Cell-Surface Localization and Signaling of GPCRs

The majority of the proteins described above only modulate the expression of GPCRs. However, a few GPCRs-interacting proteins have shown to exert remarkable effects not only on the expression of GPCRs, but also on the pharmacology of these receptors (Table 2, Figure 2). Those effects are so profound sometimes, that these interacting proteins are considered as co-receptors. These specific interacting proteins bring another level of regulation where a single GPCR can display two distinct functions depending upon its interaction with distinct accessory proteins.

### 3.1. RAMPs, Co-Receptors of the Calcitonin Receptor Family

The first GPCR-interacting protein family that influenced both the traffic and the pharmacological properties of a GPCR are the receptor activity-modifying proteins (RAMPs). The RAMPs are single pass transmembrane proteins initially identified as molecular partners of the calcitonin receptor-like receptor (CLR, [80]). Agonists of CLR belong to the calcitonin peptide family that includes calcitonin (CT), islet amyloid polypeptide (IAPP, also known as amylin), calcitonin gene-related peptide (CGRP), adrenomedullin (ADM) and ADM-2 (also known as intermedin). This family of peptides exhibits similar biological properties on a wide range of tissues. Some of them are involved in diverse pathologies, including diabetes (IAPP, [103]), migraine (CGRP, [104]) and osteoporosis (CT, [105]). They all signal through either the calcitonin receptor (CTR) or CLR. Both GPCRs belong to the secretin family (also known as class B) [1]. CTR was first cloned in 1991 [106], shortly followed by the cloning of CLR [107]. CLR was first believed to be a receptor for either CGRP or IAPP receptor, peptides that until then, had unknown receptors [107]. However, CLR was then considered an orphan receptor, as functional experiments failed to demonstrate that any peptides of the calcitonin peptide family had an activity when CLR was transfected into COS-7 cells [108]. It was a year later that evidence of CGRP binding and CGRP-dependent cAMP activation was observed in HEK cells transfected with CLR [109]. However, the major breakthrough occurred two years later, when it was shown that when RAMPs were necessary to confer receptor function to CLR [80]. The RAMP family comprises three proteins named RAMP1-3, that share ~30% homology and consist of a single transmembrane domain with a large extracellular *N*-terminal and a short intracellular *C*-terminal [80]. If expressed on their own, neither RAMPs nor CLR traffic to the cell-surface and are retained in ER. It is only when CLR and RAMPs are co-expressed do they efficiently traffic to the cell surface [80,110]. RAMP1 is required to promote terminal glycosylation of CLR, whereas RAMP2 and 3 only promote core glycosylation of CLR [80,111,112]. Beyond the roles of RAMPs in protein trafficking and glycosylation of CLR, RAMPs are also responsible for the ligand binding specificity of the receptor. Indeed, the *N*-terminal of RAMPs lies close and forms the ligand binding pocket with CLR [112]. When CLR associates with RAMP1, it forms a high affinity receptor for CGRP (CGRP receptor). Alternatively, when CLR associates with either RAMP2 or 3, they form two distinct high affinity receptors for ADM (termed AM_1_ and AM_2_ receptors, respectively) [80,113]. It is also interesting to note that RAMPs influence agonist-induced internalization of CLR. Sequence analysis indicates that RAMP3, but not RAMP1 or 2, possesses a PDZ domain (a protein-protein binding domain, see Section 3.5) in its *C*-terminal that binds to NSF and promote the recycling of AM_2_ receptor after internalization [114]. Moreover, another study has shown that CGRP receptor can either recycle or be down-regulated depending on the duration of the stimulus [115].

Unlike CLR, CTR traffics to cell-surface when expressed alone and forms a fully functional receptor responsive to CT [106]. However, RAMPs have been shown to also interact with CTR [81,82], where its association with RAMPs forms three different IAPP receptors (termed amylin_1–3_ receptors). A unique receptor for ADM2 has not yet been defined; however, ADM2 can activate CGRP and AM_1–2_ receptors, as well as IAPP receptors. Although, the highest affinity receptor observed for ADM2 is AM_2_ receptors [116]. Therefore, RAMPs enable a complex system of pharmacological diversity with only two GPCRs. Moreover, the promiscuity of the calcitonin peptide family for the receptors of the CTR, CLR and RAMP system could also explain the observed similarity in the biological effects of these peptides [82]. It certainly suggests that these receptors must be tightly regulated in order to present the correct cellular phenotype.

### 3.2. RAMPs, Beyond the Calcitonin Receptor Family

Studies have established that RAMPs are more widely distributed than both CTR and CLR [80,117–119], suggesting additional potential roles for RAMPs with other GPCRs. Further, studies with *Ramp2*^(−/−)^ mice have revealed distinct roles for these proteins. For example, knockout of *Ramp2* is embryonic lethal, with the mice suffering from extreme generalized edema formation and blood vasculature abnormalities, similar to *Adm*^(−/−)^ and *Clr*^(−/−)^ mice [120–123]. Moreover, *Ramp2*^(−/+)^ mice showed more extended phenotypes than *Clr*^(−/+)^ *mice*, suggesting that RAMP2 has additional functions. On the other hand, *Ramp1*^(−/−)^ and *Ramp3*^(−/−)^ mice are viable and display distinct phenotypes. *Ramp1*^(−/−)^ mice have hypertension and display dysregulated immune responses, whereas *Ramp3*^(−/−)^ mice appear normal until old age, at which point they have decreased weight, indicating defects in metabolism [122,124]. Together, these facts imply that RAMPs could potentially interact with other GPCRs. Thus, studies have sought to investigate this possibility. It is now known that RAMPs interact with at least four other members of the secretin GPCR family. All three RAMPs have been shown to bind to VPAC_1_ receptor (a receptor for vasoactive intestinal peptide (VIP) and pituitary adenylate cyclase-activating polypeptide), whereas RAMP2 also binds to parathyroid hormone 1 (PTH1) receptor and glucagon receptor and RAMP3 also binds to PTH2 receptor [83]. No evidence had yet been found for interactions between RAMPs and VPAC_2_ or either the glucagon-like peptide 1receptor or glucagon-like peptide 2 receptors. However, unlike to CLR, expression of RAMPs did not affect the cell-surface localization of VPAC_1_ receptor or binding of VIP to the receptor. VPAC_1_ receptor is known to signal through multiple G proteins, leading to cAMP production and activation of PLC and subsequent mobilization of intracellular calcium [125]. Quantification of these second messengers indicated that RAMP2 in particular, increased the hydrolysis of phosphatidylinositol phosphates, without affecting cAMP production. These results suggest that RAMP2 is able to promote coupling of VPAC_1_ receptor with Gα_q_-subunit [83]. The effect of RAMPs on the signaling properties of PTH1, PTH2 and glucagon receptor remains to be determined.

Although experiments first showed that RAMPs did not interact with VPAC_2_ receptor [83], a different line of experimentation observed that expression of VPAC_2_ receptors appeared to enhance the cell-surface expression of all three RAMPs, without modification of its own trafficking [84]. Further, VPAC_2_ receptor-mediated cAMP production did not show any differences when RAMPs were co-transfected. In accordance with this, co-expression of RAMPs had no effects on G_s_ coupling. However, expression of RAMP1 and 2 significantly increased basal coupling of Gα_i/o/t/z_-subunit to the receptor, the functional relevance of this remains unclear. The same study also revealed that RAMP2 could promote trafficking of corticotropin-releasing factor receptor 1 (CRF_1_) to the cell-surface. Similar to the VPAC_2_ receptor, RAMP2 had no effect on the coupling of CRF_1_ receptor to Gαs-subunits, but did enhance coupling to G_i/o/t/z_, G_q/11_ and G_12/13_ subunits. They also determined that CFR_1_-induced increases intracellular calcium were dependent on G_i/o/t/z_ subunits and relied on the entry of calcium from outside of the cell. These effects on calcium levels were observed when corticotrophin releasing factor (CRF) and urocortin were used as agonists and not sauvagine [84], a potent vasoactive peptide isolated from frog skin [126]. This study concluded by examining the CRF responses in *Ramp2*^(−/+)^ mice. The researchers observed blood levels of adrenal corticotrophic hormone (ACTH) were reduced in response to CRF, compared to *Ramp2*^(+/+)^ mice.

These results suggest that RAMPs play an important role in the regulation and signaling of many GPCRs belonging to the secretin GPCR family. However, RAMPs have also been shown to regulate a receptor of the glutamate GPCR family. The Ca^2+^-sensing receptor is retained in ER when transfected in COS-7 cells, but co-transfection with RAMP1 or 3, not only promotes trafficking of Ca^2+^-sensing receptor to the cell-surface, but also similar to CLR, induces terminal glycosylation of the receptor [85]. RAMP2 had no effect on the trafficking of this receptor.

Overall, there is an abundance of evidence showing that RAMPs enable trafficking as a chaperone and also participate in the signaling of diverse set of GPCRs. There may yet be other unknown interactions of RAMPs with GPCRs, but there is still much to learn from those interactions that we discussed above that may lead to the discovery of new therapies for diseases such as migraine or diabetes.

### 3.3. MRAPs and Their Vital Role in Stress

Stress plays an important part in animal survival. It is defined by the real or perceived threat to homeostasis. Stress stimuli promote release of glucocorticoids from the adrenal glands, which induce a wide range of biological responses that include increased cardiovascular tone, respiratory rate, intermediate metabolism, immunity and inhibition of general vegetative functions such as feeding, digestion, growth and reproduction [127]. Due to the effects of the glucocorticoids on the body, dyregulation of systems that regulate their release can lead to health threats such as psychiatric disorders (e.g., depression and schizophrenia), Cushing’s syndrome and sepsis [128] and therefore is tightly regulated, mainly by the hypothalamic-pituitary-adrenal axis. Stress leads to the activation of the hypothalamic paraventricular nucleus, which promotes the secretion of CRF and arginine vasopressin into the hypophyseal portal circulation [129]. Both CRF and arginine vasopressin then trigger the release of ACTH [130]. ACTH is derived from the cleavage of its precursor proopiomelanocortin together with α-, β- and γ-melanocyte-stimulating hormones (MSHs) and endorphin [131,132]. Once released in the circulation, ACTH acts mainly on the adrenal gland to stimulate glucocorticoids hormone production.

Signaling of ACTH and MSHs is mediated by melanocortin receptors (MCRs), a subfamily of the rhodopsin GPCR family [133,134]. There are five known members of MCRs, which present different tissue distribution and specificity of ligand [135]. All MCRs respond to all proopiomelanocortin-derived hormones (*i.e*., ACTH and α-, β- and γ-MSHs), albeit with different affinities. However, MC2R only responds to ACTH [133]. MC2R is predominantly expressed in the adrenal gland, where ACTH-dependent activation promotes production and secretion of glucocorticoid hormones [134]. Failure of MC2R to respond to ACTH causes a hereditary disease, called familial glucocorticoid deficiency (FGD) [136]. This is a rare disorder caused by a single point mutation in MC2R, resulting in loss of function. The loss of function is brought about by the receptor’s failure to traffic appropriately to the cell-surface [137]. FGD patients generally present profound hypoglycaemia or overwhelming infections and the disease normally is lethal in early childhood if left untreated [138]. Mutations in MC2Rs account for approximately 25% of all FGD cases and are defined as FGD type 1 [139]. Similarly to ORs, the study of MC2R in heterologous cells was difficult also because of its lack of expression at the cell-surface. Transfection of MC2R alone resulted in its retention in ER [140]. Therefore, investigators hypothesized that MC2R traffic to the plasma membrane was regulated by additional factors.

FGD patients that had a normal *MC2R* gene, but still lacked cell-surface expression were identified. The patients’ DNA was genetically screened and a number of nonsense splice site mutations were identified in *C21ORF61* gene [86]. This gene was found to encode a small transmembrane protein that is highly expressed in the adrenal gland. Furthermore, once cloned, it was shown to interact with MC2R and thus was named melanocortin 2 receptor accessory protein 1 (MRAP1) [86]. Co-transfection of MC2R and MRAP1 in CHO cells results in the translocation of MC2R to the cell-surface [86]. MRAP consists of 172 amino acids containing a highly conserved *N*-terminal, a single transmembrane domain and a less conserved *C*-terminal domain [86]. MRAP1 presents a unique topology, by forming an antiparallel homodimer [141], which then forms a stable heterodimer with MC2R at the cell-surface [87]. Interestingly, the *N*-terminal domain of MRAP1 comprises a tyrosine-rich region that is also found in the *N*-terminal of REEP1, suggesting that this may be a common feature involved in GPCR trafficking [142]. Studies have investigated the effects of mutating residues within this region. The studies showed that although MRAP1 mutants can still promote traffic of MC2R to the cell-surface, they result in the inability of the receptor complex to respond to ACTH, suggesting that MRAP1 influences the ligand binding and/or specificity of the MC2R [87].

Recently, a homologue of MRAP was identified and named MRAP2. MRAP2 is more conserved between species than MRAP1 and also interacts with MC2R to promote its traffic and influence its signaling [88]. Both MRAP1 and 2 interact with the other MCRs, however the effects on function are different to those observed for MC2R. For example, co-transfection of MC4R or MC5R with MRAP1 and/or MRAP2 in CHO cells results in decrease of cell-surface expression and reduction of [Nle^4^, dPhe^7^]-α-MSH-mediated signaling of these receptors. MRAPs have no effect on cell-surface localization of MC3R, but negatively affect signaling [88]. This data shows that MRAPs are important regulators of MCRs function and especially for MC2R in the context of FGD. However, more studies are required to clearly assess the role of MRAPs *in vivo*. Likewise, it would be interesting to investigate if MRAPs are able to interact with other GPCRs, which given their wide distribution, is enticing to hypothesize.

### 3.4. DRiP78, a Regulator of the G Protein•GPCR Complex

An important step in the expression of transmembrane proteins is their exit from the ER. Generally, proteins to be exported possess an ER-export motif that is recognized by protein cargos that belong to the coat protein complexes. Coat protein complexes then polymerize and form a “bud” that traffics to the cell-surface [143]. Several ER-export sequences have already been identified for membrane proteins, such as “DxE” for the vesicular stomatitis virus glycoprotein [144] and “FF” for ER-Golgi intermediate compartment-53 (ERGIC-53) [145] and the p24 receptor family [146], where mutation of these motifs significantly impairs anterograde transport. In comparison, far less is known about ER-export motifs present in GPCRs.

A study on the D_1_ receptor, not only identified the ER-export motif “FxxxFxxxF” present in its *C*-terminal tail, but also revealed the specific binding of an ER-membrane-associated protein named dopamine-receptor-interacting protein of 78 kDa (DRiP78) [89]. DRiP78 comprises of two predicted transmembrane domains that co-localize in the ER. Mutation of the ER-export motif leads to retention of D_1_ receptor to ER and subsequently, decreases the dopamine-induced cAMP production [89]. DRiP78 (also known as DNAJC14), belongs to the HSP40 family chaperone proteins that contain a J-domain, important for the recruitment of HSP70 [10,147]. Given that the ER-export motif found in D_1_ receptor was also conserved in several other GPCRs and DRiP78 is widely expressed throughout the body, it was not surprising that DRiP78 also observed to regulate other GPCRs. DRiP78 negatively regulates the cell-surface expression of the muscarinic acetylcholine 2 receptor [89] and the adenosine 1 receptor [90]. Conversely, the angiotensin II receptor type 1 (AT_1_ receptor) cell-surface expression is enhanced by DRiP78 co-transfection [91]. DRiP78 has also been shown to bind to the β_2_-adrenoceptor, but its function is mainly involved in the assembly of the Gβγ protein subunits of this receptor [92]. Association of GPCRs with βγ-subunits occurs in the ER, before any interaction with the Gα-subunits which is thought to occur outside the ER, but before the Golgi [148]. DRiP78 has been shown to specifically and selectively bind to certain γ-subunits and not α- or β-subunits. The GPCR•DRiP78•γ-subunit complex protects the γ-subunit from degradation until the stable binding with the β-subunit is complete [92]. Similarly, another protein phosducin-like protein has been shown to also regulate the G protein assembly by interacting with β-subunits [149,150]. Interestingly, phosducin-like protein has been found to interact with DRiP78, suggesting that both proteins form a complex to promote the association of the βγ-subunit [92].

Recently, a study from the same group revealed that DRiP78 also regulates the trafficking of the chemokine receptor 5 (CCR5). Overexpression of DRiP78 resulted in the retention of the receptor in ER and modulated the association of the βγ-subunit to the receptor [93]. Altogether, DRiP78 seems to act as quality control point, where DRiP78 ensures correct association of the G protein complex with its GPCR before the export from the ER. However, as previously mentioned DRiP78 is also able to enhance cell-surface expression of AT_1_ receptor [91], suggesting that DRiP78 possesses additional function. Thus, further investigations are necessary to fully understand the role of DRiP78 in the regulation of GPCRs.

### 3.5. The PDZ-Domain Containing Family of Proteins

This family of proteins contains one or more PDZ domains, named after the discovery of the first three proteins containing this domain: Postsynaptic density protein 95, Drosophila disc and Zonula occludens protein 1 [151]. Although, PDZ proteins do not seem to function as the other proteins described above, they are to date, the best characterized of all the GPCRs-interacting proteins that modulate GPCR signaling and desensitization (reviewed in [152,153]). For example, the Na^+^/H^+^ exchanger regulatory factor (NHERF) protein family, also known as Ezrin-Radixin-Moesin-binding phosphoprotein 50, are scaffold proteins that link membrane proteins to the cytoskeleton and are also involved in the signaling transmission [154]. A yeast two-hybrid assay showed that NHERF2 binds strongly to the *C*-terminal tail of PTH1 receptor through its two PDZ domains [94]. Normally, the PTH1 receptor signals through the activation of adenylate cyclase via Gα_s_-subunits; however, upon NHERF2 interaction, PTH1 receptor preferentially couples to Gα_q_-subunit, induces activation of the PLC and mobilization of intracellular calcium [94]. Similar effects were reported for NHERF1 that also switches the coupling of PTH1 receptor from Gα_s_- to Gα_q_-subunit [95]. In fact, other studies reported that NHERF1-2 can bind Gα_q_-subunit, PLC and PKC [100,155,156], suggesting that NHERFs act as a scaffold protein bringing the effectors of the pathway together to facilitate the signal transmission. NHERF2 has also been shown to bind to the lysophospholipid receptor 2, [99], P2Y_1_ purinergic receptor [100] and the metabotropic glutamate 5 (mGlu_5_) receptor [101], where NHERF2 also potentiates the receptor-induced PLC activation, prolonging the intracellular Ca^2+^ mobilization.

Interestingly, the role of NHERF is not only limited to the modulation of GPCR signaling. Indeed, NHERF1 has been found to bind β_2_-adrenoceptors and κ receptors to promote efficient recycling of these receptors [157,158]. The PDZ domain found in the *C*-terminal tail of these receptors, when inserted into the *C*-terminal tail of the δ receptor, is sufficient to induce recycling of this receptor that normally undergoes efficient down-regulation [159]. Moreover, NHERF1 also regulates the internalization and desensitization of the PTH1 receptor, by inhibiting its interaction with β-arrestin2 [96,97]. After stimulation with parathyroid hormone, NHERF1 dissociates from the PTH1 receptor, an action, which promotes sequestration of PTH1 receptor within cell-surface microdomains. These microdomains are associated with the actin cytoskeleton and clathrin and lead to the stabilization of the PTH1 receptor at the cell-surface [98]. NHERF1 also plays an important role in the internalization of the P2Y_12_ receptor. In the absence of agonist NHERF1 binds to the *C*-terminal tail of the P2Y_12_ receptor, but following exposure to agonist this direct interaction is broken by the recruitment of β-arrestins to the GPCR. NHERF1 subsequently binds to β-arrestins promoting the internalization of P2Y_12_ receptor [160].

Another PDZ domain-containing protein with a role in GPCR regulation is Disks large homolog 1 (DLG1; also known as synapse-associated protein 97) [161–163]. DLG1 possesses three PDZ domains and it has recently been demonstrated to interact with the PDZ domain found within the *C*-terminal tail of the CRF_1_ receptor [102]. Knockdown of DLG1 did not affect agonist-stimulated cAMP generation, but markedly attenuated mitogenic signaling. Furthermore, DLG1 did not efficiently internalize with the CRF_1_ receptor, which perhaps implies a role for β-arrestins in regulating the interaction of DLG1 with the CRF_1_ receptor, similar to that of NHERF1 and the P2Y_12_ receptor.

PDZ domain-containing proteins regulate many cellular functions, including modulation of GPCR trafficking and signal transduction [153]. There are about 400 proteins containing PDZ domains in humans, which may potentially have a role of GPCR regulation [164]. This type of interaction brings another level of signaling diversity by allowing one receptor to signal through different Gα-subunits or by regulating its cell-surface expression, depending on the cell type. Further understanding the interactions between GPCRs and PDZ domain-containing proteins will unravel new mechanisms that can be targeted for therapeutic use.

## 4. Interacting Proteins That Modulate GPCR Signaling

It is often observed that GPCRs can couple to different G proteins complex depending on the cell-type, thereby modifying their signaling pathways as described previously with the PDZ proteins [165–167]. Further studies have provided evidence that other proteins are able to modulate the G protein-mediated signaling of GPCRs (Table 3, Figure 2).

### 4.1. RCP and CLR: Proof Three Is not a Crowd

There is evidence of another protein that associates with CGRP and AM_1–2_ receptor. Receptor component protein (RCP) was first identified in 1996 through its ability to promote the CGRP-mediated responses in *Xenopus laevis* oocytes [168]. RCP was specific for CGRP and AM_1–2_ receptor, as it had no effect on the signaling of receptors for CT, IAPP, neuropeptide Y, VIP and β-endorphin [168,176]. RCP appeared to be highly conserved in mice, rabbits and humans (~82% homology at protein level) [177,178]. In parallel, the cloning and expression of CLR in HEK cells showed evidence of CGRP binding and CGRP-induced cAMP production [109], but not in COS-7 cells [179], suggesting the presence of a component in HEK cells that makes CLR a functional receptor. However, when CLR was transfected with RAMPs only in COS-7 cells, it yields a functional receptor [180]. Later studies showed that in fact RCP was endogenously found in COS-7 cells [181]. Additionally, the distribution of RCP expression in the rat central and peripheral nervous system correlates with that of CGRP [182]. The use of an RCP antisense construct transfected in NIH 3T3 cells has been shown to reduce ADM-induced activation. However, it had no effect on ADM binding or receptor expression at the cell-surface [176,181], suggesting that RCP does not act as a chaperone to target CLR to the cell-surface, but rather it facilitates efficient coupling of the receptor to the cellular signaling pathways. A recent study reported that RCP physically interacts with the second intracellular loop of CLR and inhibition of this interaction leads to decreased CGRP-induced signaling [183]. However, the real implications of RCP on the functional complex receptor remain to be determined, as a study in yeast strongly suggests that RAMPs alone are sufficient to confer functional activity to CGRP and AM_1–2_ receptors [184].

### 4.2. Calmodulin

Calmodulin (CaM) is a small (17kDa) highly conserved protein involved in sensing Ca^2+^ ions. It is ubiquitously expressed and regulates a diverse set of cellular events. CaM was first identified as an enhancer of the activity of cyclic nucleotide phosphodiesterases (enzymes that hydrolize cAMP or cyclic guanosine monophosphate) [185,186]. CaM belongs to the EF-Hand-containing protein family [187]. The association of Ca^2+^ with CaM promotes a conformational change in CaM that allows CaM to bind to variety of proteins [188]. Not surprinsingly, CaM have been shown to interact with many GPCRs, with a plethora of effects on the GPCRs signaling. For example, agonist-mediated CaM activation is involved in the inhibition of G protein activity, either by impairing the coupling of Gα_q_-subunit with the receptor, as for the μ receptor [169], the D_2_ receptor [170] and the 5-HT_2A_R [171] or by blocking downstream effectors of G protein signaling, such as PLC for the PTH1 receptor [172]. Interestingly, CaM has also been showed to bind to the V_2_ receptor, a receptor that usually couples to Gα_s_-subunits. However, CaM did not modify V_2_ receptor-mediated cAMP production, but enhanced the intracellular Ca^2+^ mobilization [173]. Another role for CaM is in the regulation of GPCR phosphorylation. CaM, when associated with Ca^2+^, can bind to the *C*-terminal tail of the mGlu_7_ receptor, thereby preventing the ability of PKC, cAMP-dependent protein kinase and cyclic guanosine monophosphate-dependent protein kinase to phosphorylate a serine residue close to the CaM binding site [174,175]. However, the effects of CaM binding to the receptor or the phosphorylation of the *C*-terminal remain unknown.

Overall, CaM seems to play an important regulatory role in the Ca^2+^-dependent signaling of GPCRs and the mechanisms regulating the GPCR•CaM interaction may offer targets to regulate GPCR-dependent signaling. However, the mechanisms that regulate CaM are still poorly understood and much work remains to be done to fully understand its role.

## 5. Conclusions

GPCRs are key mediators of cellular homeostasis and must be carefully regulated to avoid any unbalance between signaling and desensitization that may result in the manifestation of disease. Although, the desensitization and down-regulation of GPCRs have been highly documented, mechanisms regulating the trafficking of GPCRs to the cell-surface remain poorly understood, but are nonetheless important. Indeed, mutations within GPCR sequences can cause misfolding, alter location and lead to retention in ER and/or degradation of the receptor. These events are involved in diseases and disorders, such as diabetes, retinitis pigmentosa and hypogonadotropic hypogonadism [189]. Accumulating evidence has shown that certain GPCRs require interactions with specific interacting proteins. These “so-called” molecular chaperones play critical roles in the folding, maturation, traffic to and from the cell-surface and even participate in the agonist binding properties of GPCRs. Additionally, several protein families have been found to modulate the G protein-dependent signaling of GPCRs. GPCRs remain important drug targets and one of the major problems of today’s therapeutic is the lack of specificity, often resulting in determinental side effects or lack of efficacy. By these targeting interacting proteins (which often work through different mechanims, dependent on cell-type), these issues may be avoided and so provide an effective and safer strategy for the treatment of GPCR-driven diseases.

## Figures and Tables

**Table 1.** Interacting proteins that influence cell-surface localization of G protein-coupled receptors (GPCRs).

GPCR(s)	Interacting protein(s)	Function(s)	References
Rh1 receptor	ninaA	Promote correct folding and cell-surface expression	[29–31]
Red/green opsins	RanBP2	Potentially promote correct folding and cell-surface expression	[32]

ORs	RTP1-2 and REEP1	Promote cell-surface expression. Co-localize at the cell-surface with ORs	[33]
TAS2Rs	RTP3-4	Promote cell-surface expression	[34]
δ-μ receptor heterodimer	RTP4	Promote cell-surface expression	[35]

5-HT_1B_R and 5-HT_4_R	S100-A10	Promote cell-surface expression	[36,37]

δ receptor	GASPs	Promote down-regulation of the receptor	[38]
β_2_-adrenoceptor	GASPs	Promote down-regulation of the receptor	[38]
D_2_ receptor	GASPs	Promote down-regulation of the receptor	[39]
μ receptor	GASPs	Promote down-regulation of the receptor	[40]
κ receptor	GASPs	Promote down-regulation of the receptor	[40]
β_1_-adrenoceptor	GASPs	Promote down-regulation of the receptor	[40]
CTR	GASPs	Promote down-regulation of the receptor	[40]

PAR_1_	SNX-1	Promote internalization of the receptor	[41,42]
oxytocin receptor	SNX-1	Promote internalization of the receptor	[43]
δ receptor	SNX-1	Promote internalization of the receptor	[43]
Neurokinin 1 receptor	SNX-1	Promote internalization of the receptor	[43]

**Table 2.** Interacting proteins that influence both cell-surface localization and signaling of GPCRs.

GPCR	Interacting protein(s)	Function(s)	References
CLR	RAMPs	Promote cell-surface expression, receptor glycosylation and induce ligand binding specificity	[80]
CTR	RAMPs	Induce ligand binding specificity to IAPP	[81,82]
VPAC_1_ receptor	RAMPs	Increase the coupling of Gα_q_-subunit with the receptor	[83]
PTH1 receptor	RAMP2	?	[83]
Glucagon receptor	RAMP2	?	[83]
PTH2 receptor	RAMP3	?	[83]
VPAC_2_ receptor	RAMPs	Increase the coupling of Gα_i/o_-subunit to the receptor, but RAMP3	[84]
CRF_1_ receptor	RAMP2	Promote cell-surface expression and increase Ca^2+^ mobilization	[84]
Calcium Sensing receptor	RAMP1 and 3	Promote cell-surface expression and receptor glycosylation	[85]

MCRs	MRAPs	Regulate differently the cell-surface expression depending on the MCR and also can modulate MCR-mediated signaling	[86–88]

D_1_ receptor	DRiP78	Retain receptor in ER	[89]
Muscarinic acetylcholine 2 receptor	DRiP78	Retain receptor in ER	[89]
Adenosine 1 receptor	DRiP78	Retain receptor in ER	[90]
AT_1_ receptor	DRiP78	Promote cell-surface expression	[91]
β_2_-adrenoceptor	DRiP78	Promote the coupling of the βγ-subunit	[92]
Chemokine receptor 5	DRiP78	Retain receptor in ER and promote the coupling of the βγ-subunit	[93]

PTH1 receptor	NHERF1-2	Switch the coupling of Gα_s_ to Gα_q_ of the receptor.	[94,95]
		Inhibit internalization and desensitization of the receptor	[96–98]
LPA_2_ receptor	NHERF2	Potentiate PLC signaling	[99]
P2Y_1_ receptor	NHERF2	Potentiate PLC signaling	[100]
mGlu_5_ receptor	NHERF2	Potentiate PLC signaling	[101]

CRF_1_ receptor	DLG1	Inhibit receptor agonist-induced internalization and promote	[102]
		ERK1/2 signaling	

**Table 3.** Interacting proteins that influence signaling of GPCRs.

GPCR	Interacting protein	Function(s)	References
CLR	RCP	Enhance CGRP- and ADM-mediated signaling	[168]

μ receptor	CaM	Impair G protein coupling	[169]
D_2_ receptor	CaM	Impair G protein coupling	[170]
5-HT_2A_R	CaM	Impair G protein coupling	[171]
PTH1 receptor	CaM	Inhibit Gα-mediated PLC activation	[172]
V_2_ receptor	CaM	Enhance Ca^2+^ mobilization	[173]
mGlu_7_ receptor	CaM	Regulate GPCR phosphorylation	[174,175]

## Abbreviations

5-HT: 5-hydroxytryptamine
5-HTR: 5-HT receptor
δ, κ: μ receptors
δ, κ: μ opioid receptors
ACTH: adrenal corticotrophic hormone
ADM: adrenomedullin
AM receptor: adrenomedullin receptor
AT_1_ receptor: angiotensin II receptor type 1
CaM: calmodulin
cAMP: cyclic AMP
CGRP: calcitonin gene-related peptide
CLR: calcitonin receptor-like receptor
CRF_1_ receptor: corticotropin releasing factor receptor 1
CRF: corticotropin releasing factor
CT: calcitonin
CTR: calcitonin receptor
DLG1: disks large homolog 1
D receptor: dopamine receptor
DRiP78: dopamine-receptor-interacting protein of 78 kDa
ER: endoplasmic reticulum
ErbB1: epidermal growth factor receptor
FGD: familial glucocorticoid deficiency
GASP: GPCR-associated sorting protein
GPCR: G protein-coupled receptor
GPK: GPCR kinase
HSP: heat shock protein
IAPP: islet amyloid polypeptide
MCR: melanocortin receptor
MRAP: melanocortin 2 receptor accessory protein
MSH: melanocyte-stimulating hormone
mGlu receptor: metabotropic glutamate receptor
NHERF: Na^+^/H^+^ exchanger regulatory factor
ninaA: neither inactivated nor after potential A
ODR-4: odorant protein 4
OR: odorant receptor
PAR: proteinase-activated receptor
PDZ: postsynaptic density protein 95, drosophila disc and zonula occludens protein 1
PKC: protein kinase C
PLC: phospholipase C
PTH: parathyroid hormone
RAMP: receptor activity-modifying protein
RanBP2: Ran binding protein 2
RCP: receptor component protein
REEP: receptor expression enhancing protein
Rh1: rhodopsin 1
RTP: receptor transporting protein
SNX: sorting nexin
VIP: vasoactive intestinal peptide
V_2_ receptor: vasopressin 2 receptor
VPAC receptor: VIP and pituitary adenylate cyclase-activating polypeptide receptor
Yip: Ypt-interacting protein
